# Retinal venular vessel diameters are smaller during ten days of bed rest

**DOI:** 10.1038/s41598-023-46177-x

**Published:** 2023-11-07

**Authors:** Adam Saloň, Göktuğ Mert Çiftci, Damir Zubac, Boštjan Šimunič, Rado Pišot, Marco Narici, Per Morten Fredriksen, Benedicta Ngwenchi Nkeh-Chungag, Harald Sourij, Omar Šerý, Karin Schmid-Zalaudek, Bianca Steuber, Patrick De Boever, Nandu Goswami

**Affiliations:** 1 Division of Physiology & Pathophysiology, Otto Loewi Research Center for Vascular Biology, Immunology, and Inflammation, Medical University of Graz, Graz, Austria; 2 Research Unit “Gravitational Physiology and Medicine”, Physiology, Otto Loewi Research Center, Medical University of Graz, Neue Stiftingtalstraße 6/D.05, 8010 Graz, Austria; 3 Department of Psychiatry, University Hospital Münster, Münster, Germany; 4 Institute for Kinesiology Research, Science and Research Centre Koper, Koper, Slovenia; 5 Department 1 of Internal Medicine, Centre for Integrated Oncology, Aachen, Bonn, Cologne, Düsseldorf, University Hospital of Cologne, Cologne, Germany; 6 Department of Biomedical Sciences, University of Padua, Padua, Italy; 7 Faculty of Applied Ecology, Agricultural Sciences and Biotechnology, Inland Norway University of Applied Sciences, Lillehammer, Norway; 8 Department of Biological and Environmental Sciences, Faculty of Health Sciences, Walter Sisulu University PBX1, Mthatha, 5117 South Africa; 9 Internal Medicine, Division of Endocrinology and Diabetology, Interdisciplinary Metabolic Medicine Trials Unit, Medical University of Graz, Graz, Austria; 10 Laboratory of Neurobiology and Molecular Psychiatry, Department of Biochemistry, Faculty of Science, Masaryk University, Kamenice 753/5, 625 00 Brno, Czech Republic; 11 Laboratory of Neurobiology and Pathological Physiology, Institute of Animal Physiology and Genetics, Czech Academy of Sciences, Veveří 97, 602 00 Brno, Czech Republic; 12 Centre for Environmental Sciences, Hasselt University, Hasselt, Belgium; 13 Integrative Health, Alma Mater Europaea Maribor, Maribor, Slovenia; 14 College of Medicine, Mohammed Bin Rashid University of Medicine and Health Sciences, Dubai, United Arab Emirates

**Keywords:** Cardiovascular biology, Public health, Circulation

## Abstract

Older individuals experience cardiovascular dysfunction during extended bedridden hospital or care home stays. Bed rest is also used as a model to simulate accelerated vascular deconditioning occurring during spaceflight. This study investigates changes in retinal microcirculation during a ten-day bed rest protocol. Ten healthy young males (22.9 ± 4.7 years; body mass index: 23.6 ± 2.5 kg·m^–2^) participated in a strictly controlled repeated-measures bed rest study lasting ten days. High-resolution images were obtained using a hand-held fundus camera at baseline, daily during the 10 days of bed rest, and 1 day after re-ambulation. Retinal vessel analysis was performed using a semi-automated software system to obtain metrics for retinal arteriolar and venular diameters, central retinal artery equivalent and central retinal vein equivalent, respectively. Data analysis employed a mixed linear model. At the end of the bed rest period, a significant decrease in retinal venular diameter was observed, indicated by a significantly lower central retinal vein equivalent (from 226.1 µm, CI 8.90, to 211.4 µm, CI 8.28, p = .026), while no significant changes in central retinal artery equivalent were noted. Prolonged bed rest confinement resulted in a significant (up to 6.5%) reduction in retinal venular diameter. These findings suggest that the changes in retinal venular diameter during bedrest may be attributed to plasma volume losses and reflect overall (cardio)-vascular deconditioning.

## Introduction

Bed rest has been prescribed to treat various medical conditions throughout the twentieth century^1–3^. However, in the current medical practice, daily activity and physical rehabilitation take precedence due to the revealed adverse effects of prolonged bed rest^1,2,4^. Nevertheless, confinement to bed is still common in care homes, where elderly or chronically ill individuals may spend extended periods bedridden for essential activities such as eating, personal care, toileting, and bathing^5^. Additionally, bed rest is frequently used as a model to study the physiological effects of weightlessness during spaceflight^6–8^. One widely employed approach of bedrest confinement involves 6-degree head-down tilt bed rest, which closely mimics the cephalad fluid shifts seen in microgracvity^6–11^.

Bed rest induces significant cardiovascular changes, increasing heart rate and reducing oxygen uptake (V̇O_2_ max.)^12^. These changes result from decreased maximal stroke volume and cardiac output^12^. The reduction in stroke volume is primarily attributed to decreased venous return associated with lower circulating blood volume, reduced central venous pressure, and increased venous compliance in the lower extremities^12^. Prolonged bed rest can lead to deconditioning and plasma volume loss, potentially causing deep vein thrombosis and orthostatic hypotension^5–7,13^. Plasma volume loss is a hallmark of extended bed rest^14–19^. Investigating the physiological response to bed rest is crucial for mitigating adverse effects in medical treatments, gaining insights into aging-related deconditioning, and advancing our understanding of space physiology in humans.

Traditional methods for assessing cardiovascular physiology include blood marker evaluations, chest x-rays, ECGs, echocardiogram, exercise, or stress tests, cardiac catheterization, and CT or MRI scans. Retinal vessel analysis emerged as a non-invasive physiological technique to assess cardiovascular and cerebrovascular health^20–23^.

The retinal blood vessels originate from the ophthalmic artery, the first branch of the internal carotid artery, and changes in retinal vessel widths are proposed as surrogate markers for evaluating systemic circulation^24^. Several studies have confirmed that changes in retinal vessel caliber can predict of changes in larger vessels^25–34^. Notably, constrictions of retinal arterioles and veins is associated with future increase in blood pressure and the development of hypertension^27–29^. Additionally, constriction of retinal arterioles and dilatation of retinal venules serves as strong predictors of coronary heart disease and clinical stroke^30–34^.

The retina, like other organs in the body, has autoregulatory mechanisms to maintain adequate blood flow and oxygen delivery. During microgravity, when blood volume is pulling upwards and mean arterial pressure decreases, the retinal vessels may respond by constricting (narrowing). This constriction is an attempt to maintain sufficient perfusion pressure and oxygen supply to the retinal tissues. This is supported by limited number of studies that are available^35–39^. The exact mechanisms behind these changes are still not fully understood.

Our ten-day horizontal bed rest study involving ten healthy young men investigated the impact of vascular unloading on the microvascular health. Our hypothesis is that bed rest-induced plasma volume loss and general vascular deconditioning will affect retinal microcirculation, resulting in observable narrowing of retinal vessel diameters.

## Methodology

This horizontal bedrest study was performed at the Izola General Hospital (Izola, Slovenia). Data were collected according to the principles of good clinical practice and the WMA Declaration of Helsinki. Ethics approval was granted by the National Ethics Committee of the Slovenian Ministry of Health on July 17, 2019, under reference number 0120-304/2019/9. Participants were adequately informed about the study by the responsible hospital staff. All participants provided signed informed consent prior to study enrolment.

### Participants

Ninety-three participants were initially invited to the study. Forty-six young male participants were invited for interviews, and 16 of these met all inclusion/exclusion criteria. All participants underwent a medical examination, a physical activity questionnaire (GPAQ), body composition analysis, electrocardiography (at rest and during exercise) with blood pressure measurement, medical questionnaires, functional exercise assessment, and a dietary interview. Participants were housed in standard air-conditioned hospital rooms (five participants per room) and were under constant visual monitoring with 24-h medical care, 24-h heart rate monitoring, and physical activity measurements. During bed rest, study participants performed all daily activities in bed, with no deviations from the horizontal (supine) position allowed, and both physical activity and muscle contraction tests were not allowed during the period of bed rest. All participants received hospital meals three times daily and maintained an individually controlled equicaloric diet throughout the hospital stay. Dietary energy requirements were determined for each participant by multiplying resting energy expenditure by factors of 1.2 and 1.4 in the bed rest and outpatient phases, respectively^40^. The macronutrient content of the diet was set at 60% carbohydrate, 25% fat, and 15% protein. Sleep time was between 10:00 pm and 7:00 am. The study details are given in the publication of Monti et al.^41^.

### Study protocol

**Figure 1:**
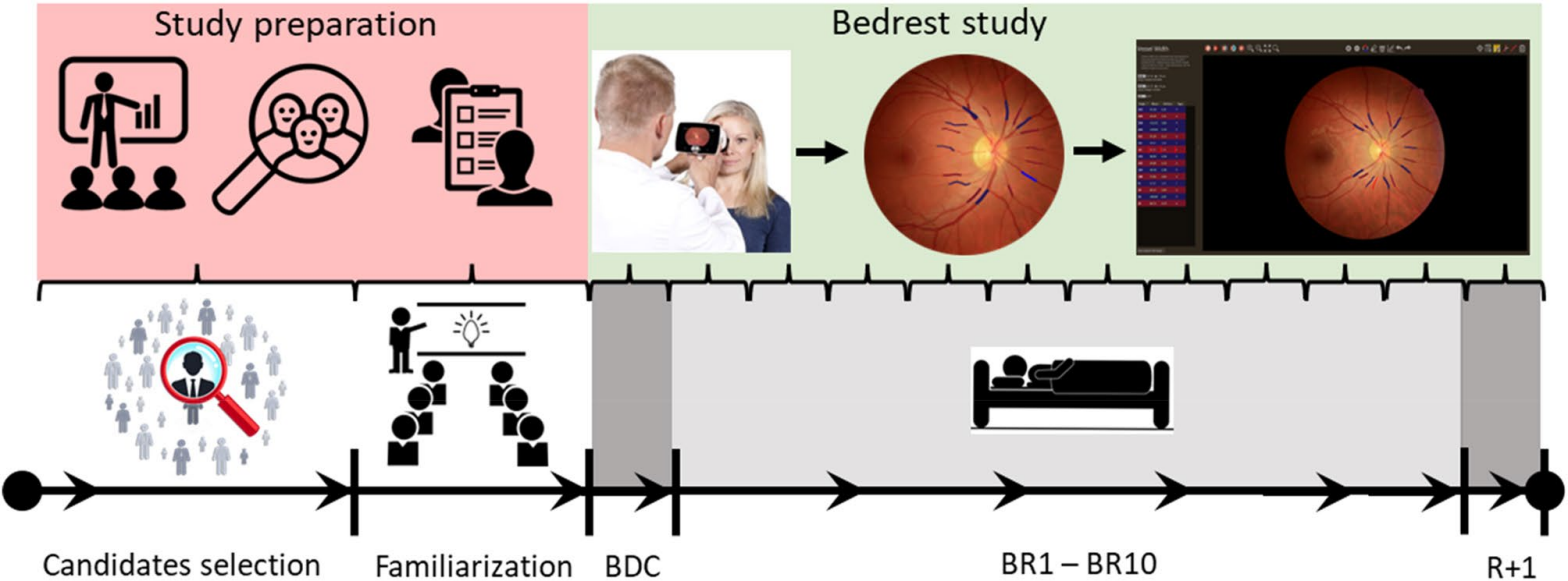
Simplified overview of the study flow including study preparation (selection of the study candidates and familiarization with the study) and bed rest study itself (*BDC* start of bed rest (baseline); *BR1-10* ten days of bed rest; *R* + *1* one day after re-ambulation (recovery)). Indicated are also the time points whent the retinal images were collected and analyzed.

The protocol included two days of familiarization with the hospital environment and diet. Baseline data collection was performed just before the start of bed rest (BDC), followed by daily data collection during the ten days of bed rest (BR1-BR10) and one day after re-ambulation (R + 1). The simplified overview of the study flow can be seen in Fig. 1. This study was part of a larger study that also included other measurements such as anthropometric characteristics, MRI, ultrasound measurements, muscle biopsies, and cardiopulmonary exercise testing^41^.

### Retinal vessel measurements

Retinal images focused on the optic disc (resolution of 1536 × 1536) were acquired from each participant's right eye by a trained person using the Optomed Aurora portable digital retinal camera (Optomed Oy, Oulu, Finland) at the same time every morning throughout the study at baseline (BDC), daily during the 10-days of bed rest (BR1 to BR10), and one day after bed rest (R + 1).

Retinal vessel analysis was conducted by a trained researcher from the Medical University of Graz, Austria, who was blinded to the study’s details. The semi-automated MONA REVA software (VITO, Mol, Belgium^42^) was utilized for this purpose. To minimize analysis variability, a single trained operator was responsible for this task. Before a retinal image can be analyzed, a scale ratio has to be determined. To determine the scale ratio, the distance between the centre of macula (fovea) and the centre of the optic disc (blind spot) must be measured. The distance must be measured in pixels. The scale ratio is calculated by dividing 4500 by the distance (in pixels) between the macula and blind spot^43^. This procedure is done in the MONA REVA software. The scale ratio, also known as the resolution number, was calculated as 6.8 um/pixel by averaging the individual resolution numbers from all retinal images. The software automatically processed retinal images, analyzing the diameters of retinal microvessels within the area between 1.5 and 5 times of the optic disc radius, ensuring consistency across all images. All vessels laying in this perifoveal area, larger than 100 µm diameter were automatically recognized and measured by the software. Subsequently, the algorithm of the software, inspired by Nguyen et al. performed automatic retinal vessel segmentation^42^. Post-processing included double thresholding, blob extraction, removal of small contiguous regions, and hole filling. Thereafter, the grader checked and corrected diameters and designations (arteriole or venule) using the MONA REVA vessel editing toolbox. The revised Parr-Hubbard-Knudtson formula, employing the six largest retinal arterioles and venules, was applied to calculate retinal microvascular parameters: central retinal artery equivalent (CRAE) and central retinal vein equivalent (CRVE)^44^.

### Statistical evaluation

**Figure 2:**
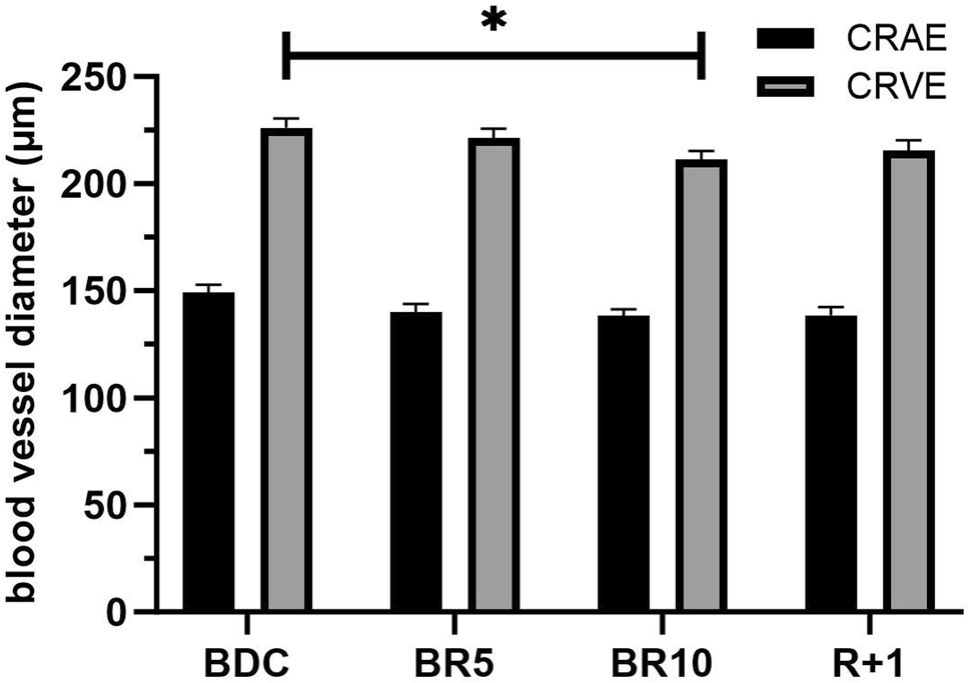
The gradual effect of 10-day of bed rest on central retinal arterial equivalent and central retinal vein equivalent (CRVE) of 10 young, healthy males. Data are shown from baseline (BDC), during bed rest (BR5 and BR10), and re-ambulation after one day (R + 1) as a mean ± 95% confidence interval.

Data were analyzed using IBM SPSS version 28.0 (Armonk, NY, USA: IBM Corp). Normality of the data was confirmed using graphical analysis of histogram and Q-Q plot, tests for kurtosis and asymmetry with a Shapiro–Wilk test. During the 12 days of measurements, missing or invalid data occasionally occurred, which was resolved using mixed linear modeling (MLM). Levene's test was used to confirm homogeneity of variance. Subjects were classified as random factors, whereas time (BDC, BR1-10, R + 1) was classified as a fixed factor. The MLM employs maximum likelihood to estimate missing data. All subjects are retained for analysis, with those having complete datasets used to estimate degrees of freedom and mean at each time point. Data from individuals with missing data are aggregated for the final averages at each data point. However, MLM compares averages of subjects with complete datasets to averages of available data from those with missing data only after confirming the randomness of the missing data. This comparison is performed at each time point. The MLM provides weighted means of the data sets that account for the effects of sample size and random/fixed factors. In addition, this model does not provide SD, only the standard error (SE) and confidence intervals (95% CI, given in Fig. 2). All statistical decisions were made at p ≤ 0.05.

## Results

Ten healthy young participants (age: 22.9 ± 4.7 years; body mass index: 23.6 ± 2.5 kg·m^–2^) took part in the study. Normotension, normal blood glucose levels, no smoking, no medication, and no occurrence of any kind of eye disease were reported.

A significant overall change in CRVE of 6.5% (Fig. 2) was seen when the start (BDC) and the end (BR10) of the bed rest period were compared. No significant differences were found in CRAE and arteriovenous ratio (AVR), which is the ratio between CRAE and CRVE.

## Discussion

The present study suggests that bed rest induces changes in microcirculation, as evidenced by retinal vessel width measurements. Bed rest led to a narrowing of retinal veins and no effect on the width of the retinal arteries (CRAE, Fig. 2). The present study hypothesizes that changes in the retinal microvascular parameters reflect changes in the cardiovascular system resulting from prolonged inactivity/bed rest^5–7,11^.

Taibbi et al. investigated the differences between a 70-day of 6° head-down tilt bed rest and 6-month space mission^45^. They found no changes in vessel diameter but did observe a decrease in retinal vessel density in astronauts. Additionally, they noticed a decrease in fractal dimension and vessel length in smaller (not larger) retinal vessels in astronauts^46^. The authors speculate that the observed decrease in vessels may be attributed to the reduction in vessel diameter, which corroborates with the results of our study in which a lower CRVE was observed. Another cross-over study examined changes in retinal vessel diameters in eleven young males (27 ± 6 years; 23.7 ± 3.0 kg m^–2^) during normoxic bed rest, hypoxic bed rest, and hypoxic ambulation^47^. The normoxic bed rest group, which most closely matches our study group observed reductions in both CRVE, consistent with our findings, and CRAE. The shorter duration of our study (10 days) compared to theirs (21 days) might explain the lack of significant results in CRAE.

Two additional studies explored short-term exposure to microgravity^48,49^. A study inducing microgravity through a parabolic flight revealed a 58% increase in intraocular pressure by and a 4% reduction in CRAE^48^. The second study, conducted by Baer and Hill, investigated the effect of passive tilting on ten healthy participants (mean age, 28.9 years; 7 men and 3 women) observed a significant 3.5% reduction in CRAE and a 3.7% increase in CRVE during head-down tilt^49^. However, unlike our study, both of these mentioned studies examined the acute effects of microgravity exposure (20 s of the parabolic flight and 3 min of head-down tilt). Therefore, their observed reduction in CRAE might be attributed to transient, acute physiological responses, rather than the gradual establishment of a new hemodynamic equilibrium typical for prolonged exposure to unloading, such as bed rest or space flight.

Small retinal vessels appear to play a significant role in regulating gravitationally related fluid shifts. The limited number of studies that are available suggest that microgravity may reduce retinal vascular density or retinal vessel diameter. Over the long term, the narrowing of the caliber of small peripheral vessels, such as retinal vessels, is expected as an autoregulatory response to hypovolemia during microgravity exposure (e.g. bed rest). Our study observed responses in retinal venules after a brief period of physical inactivity (10 days of bed rest). The smaller retinal vessel diameters may indicate the adaptation in the peripheral vascular system. The reduction in CRVE may be explained by the decrease in central blood volume due to bed rest-induced unloading, as suggested by previous research^14–19^. Enhanced peripheral vasoconstriction in individuals adapted to a microgravity environment may result from hypovolemia. Additionally, venous blood accumulation in the internal jugular vein during spaceflight could contribute to blood volume loss in the retinal vessels^50–53^. Another reason could be an increase in cerebral, respective ocular perfusion pressure because of fluid shifts. Increased blood flow in the retina triggers autoregulative constriction of retinal vessels to maintain constant blood flow. Furthermore, Du et al. reported heterogeneity in response across studies and different brain regions^54^. Additionally, reduced cerebral blood flow pulsatility during head-down tilt relative to preflight values suggests enhanced cerebral vasoconstriction, possibly due to adaptation to increased cerebral vascular pressure during microgravity^55^.

The present study did not observe any effects on retinal arteries. Previous studies have found a more significant impact on the arteries. Bed rest induces multifactorial physiological changes in vascular function and hemodynamics. The duration of exposure, partial pressures of O_2_ and CO_2_, metabolic tissue demands, the autonomic nervous system, and individual factors such as sex, age, or adaptability may all contribute to these physiological changes. When evaluating the effects of unloading, it is crucial to consider all potential factors that could influence the observed effects. Therefore, future studies should encompass the assessment of several other parameters to corroborate the microcirculatory findings of the present study.

### Limitations

The small sample size can be named as a study limitation. However, most other bed rest studies are of similar size because of challenging operational and logistic procedures. However, the strict supervision of bed rest participants reduces the experimental variability, and the repeated measures design is a strength for the statistical analysis. Because of this study design, bed rest studies typically generate reliable results^56–58^. Another limitation is that only men participated in the study. However, previous studies have shown that physiological responses to bed rest vary by sex and should be assessed individually. Future long-term studies with larger sample sizes, including female participants, are warranted to investigate the possible effects of inactivity (e.g. bed rest or microgravity) on the microcirculation. A possible limitation of our study is the 0° tilt of the bed during bed rest, whereas the usual model used to simulate weightlessness has a tilt of − 6°. However, it can be assumed that the -6° tilt only accelerates the physiological changes caused by horizontal bed rest, which can be observed in any type of bed rest^7,59^. Finally, evaluating retinal images involves a highly subjective procedure that often necessitates manual fine-tuning, introducing a potential for errors that could impact the outcomes. Nevertheless, we maintain confidence in the integrity of our results, as all data analysis was meticulously carried out by a skilled and experienced grader, ensuring consistency throughout the process.

## Conclusion and future directions

The present study investigated the effects of ten days of bed rest on retinal microcirculation parameters. A reduction of 6.5% (14.5 µm) in retinal venular diameter, summarized as CRVE, was observed at the end of the ten days compared to baseline measurements. No significant changes in arteriolar diameters were noted. The results suggest adaptation to bed rest exposure, which could be related to hypovolemia and cardiac deconditioning. Further research with a longer duration of bed rest is warranted to confirm and expand upon these findings.

Finally, a research gap exists regarding the effects gravitational changes have on retinal volumetric blood flow. Future research should, together, also consider assessment of volumetric blood flow along with retinal vessel diameters, taking into account the operational reliability of the measurements in bed rest experimental setups. For instance, Riva et al. reported average total volumetric flow rates of 33 ± 9.6 μL/minute for retinal arterial circulation and 34 ± 6.3 μL/minute for retinal venous circulation^60^, corroborating the observations of Feke et al., who noted comparable total retinal blood flow rates in both arterial and venous vessels^61^. Additionally, Garhofer et al. found that retinal venular blood flow was 42.1 ± 13.0 μL/minute, a value not significantly different from that observed in the arterioles at 43.3 ± 12.1 μL/minute^62^. Indeed future investigations of how shifts in the body fluids during central hypovolemia and/or microgravity influence retinal blood flow, using technologies such as Laser Doppler Velocimetry or Optical Coherence Tomography, could provide important information regarding retinal vasculature physiology.

## Data Availability

All data generated or analyzed during this study are included in this published article. For any specific additional inquiries or requests for further information, please contact the corresponding author of this manuscript.

## Abbreviations

CRAE: Central retinal artery equivalent
CRVE: Central retinal vein equivalent
AVR: Artery-to-vein ratio
V̇O_2_ max.: Maximal oxygen uptake
MLM: Mixed linear modelling
BDC: Baseline
BR1-BR10: First to tenth day of bed rest
R + 1: One day after re-ambulation
